# Ultrasound targeted microbubble destruction combined with Fe-MOF based bio-/enzyme-mimics nanoparticles for treating of cancer

**DOI:** 10.1186/s12951-021-00835-2

**Published:** 2021-03-31

**Authors:** Xi Xiang, Houqing Pang, Tian Ma, Fangxue Du, Ling Li, Jianbo Huang, Lang Ma, Li Qiu

**Affiliations:** 1 Department of Medical Ultrasound, Laboratory of Ultrasound Imaging Drug, West China Hospital of Sichuan University, Chengdu, 610041 China; 2 Department of Ultrasound, West China Second University Hospital, Sichuan University/West China Women’s and Children’s Hospital, Chengdu, 610041 China

## Background

Cancer is an important disease that seriously threatens human health, such as ovarian cancer, which is a salient public health concern and remains the deadliest form of gynaecological malignancy despite of its infrequent incidence [1]. It ranks the 7th most common form of cancer and the 8th leading cause of cancer-related death among women worldwide. [2, 3] Currently, the treatment of cancer includes surgery [4], chemotherapy [4], targeted therapy [5], but it is still difficult to achieve a radical cure of the cancer due to the risk of recurrence and treatment resistance [1, 4]. Therefore, there is an urgent need to find new strategies to treat cancer.

With the rapid development of nanomedicine, the application of nanoparticles in anti-tumor therapy has attracted more and more attention. Reactive oxygen species (ROS) are a group of molecules formed from incomplete reduction of oxygen, including superoxide anion (O_2_^·−^), hydrogen peroxide (H_2_O_2_), hydroxyl radical (·OH), and so on [6]. Among them, ·OH exhibits indiscriminate reactivity and is highly oxidative to all biological targets [7]. ROS is prevalent in various diseases, for instance cancer, cardiovascular diseases and neurodegenerative diseases [8]. The balance of production and removal of ROS in the niche of normal cells keeps a dynamic balance. Low concentration of ROS in cells mediates intracellular signals, while the over-expression of ROS caused cytotoxicity, leading to apoptosis or necrosis [9]. And high concentration of ROS is recognized as the hallmark of cancer cells, since it is always caused by the alterations in cellular metabolism for supporting their malignant proliferation [10]. Therefore, it is a potential therapy for combating against cancer by the abrogation of redox homeostasis [11, 12]. Due to the excellent reactivity of ·OH, the conversion of H_2_O_2_ to ·OH is expected to cause greater oxidative damage in tumor cells. In addition, the standard reduction potential *E*^0′^ (H_2_O_2_, H^+^/H_2_O, ·OH) = 320 mV from the electrochemical standpoint [13], which is also thermodynamically viable [14].

Catalysis, as one of the most significant processes occurring in human bodies persistently, helps maintain the general homeostasis [6, 15]. Catalytic chemistry has rapidly developed in recent years, and it provides feasible tools for us to harness redox reactions for biochemical applications by using nanozymes to actuate redox reactions [16]. To deal with malignant cancer with effective therapeutic outcomes and alleviate adverse biotoxicities, pathological and chemical hallmarks in the tumor microenvironment have been applied to provide distinct stimulations to initiate the nanozyme reactions [17–19]. The enzyme-mimetic reagents are able to initiate Fenton-like reactions in tumor cells, and can convert hypotoxic H_2_O_2_ into hypertoxic ·OH, resulting in oxidization and inactivation of proteins and organelles in cells abruptly [20]. Furthermore, genotoxic ROS generation, metabolic insufficiency and increased toxicity of protein can be also caused by the accumulation of the damaged proteins and organelle [21]. Finally, the toxic effect received further amplification.

Despite the concentration of H_2_O_2_ in tumor cells is slightly higher than that in normal cells [22], it is still insufficient to achieve effective conversion to hypertoxic ·OH. One solution is to increase the amount of endogenous H_2_O_2_ in tumor sites by stimulating the production in situ at specific sites. Studies have shown that [23] glucose, as a cell energy source and metabolic intermediate, plays an important role in cell proliferation and growth, especially in tumor cells. As its proliferation and invasion ability is stronger than that of normal cells, a large amount of glucose is needed to provide energy for cell metabolism. Glucose oxidase (GOx) is a kind of natural endogenous oxidoxidase, which is widely distributed in organisms. It has a unique catalytic effect on glucose and can catalyze it to produce glucose acid and H_2_O_2_. Hence, GOx can be used as an efficient H_2_O_2_ generation catalyst in tumor sites, meeting the requirements of in-situ production of H_2_O_2_. At the same time, a large amount of glucose consumption in tumor cells can also cause glucose metabolism disorder, which not only increases H_2_O_2_ level in tumor areas, but also leads to starvation therapy of tumor cells.

Enzyme-mimetic catalysts, such as alkaline metals, transition metals, lanthanoid components, have been widely used in the fields of disease diagnosis, wound disinfection, and tumor treatments [24]. And engineering and modification of these catalysts can further motivate their broad biomedical applications. The metal–organic frameworks (MOFs), which are characterized by molecular/atomic level catalytic centers, large surface area, high porosity, high loading capacity, and homogeneous structures, have emerged as one of the most promising materials for the rapid and significant design of catalysts in the past 10 years [25–27]. MOFs exhibit excellent catalytic performance and selectivity, resulting from definite advantages in maximizing up to 100% the utilization efficiency of metal atoms [28, 29].

Although significant progress has been made in recent years, the direct applications of nanozymes as biomedical agents is still facing many challenges, such as poor stability, easy to be cleared, insufficient targeting, and poor biocompatibility [30]. Therefore, to synthesize effective, accurate and biocompatible nanozymes has become the key point to anti-tumor therapy. Fe-MOF based nanozyme (FeN), such as NH_2_-MIL-88, is a nanoscale MOF with intrinsic POD activity with a promising capacity of generation sufficient amount of ·OH [14], and has been explored widely in biomedicine. It is a typical isoreticular MOF derived from trimeric secondary units with MIL-88 topology [31]. In recent years, nanoparticles coated with bionic functional membranes have been proved to be of great value in anti-tumor field [32, 33]. Bionic functional membranes can be derived from a variety of cell membranes, including erythrocyte membrane, platelet membrane, tumor cell membrane, et al. Among them, tumor cell membrane has the characteristics of self-recognition of oncogenic cell lines in vitro, good biocompatibility in vivo, and has a selective targeting homing effect on tumor cells [33]. We hypothesized that FeN combined with GOx coated with tumor cell membrane could achieve immune escape, and had high biocompatibility and targeting.

However, the tumor cell membrane coated FeN may have a larger particle size, which makes it difficult to enter the cells and play an anti-tumor role. The big challenge for the nanoparticles is to pass through the tumor cell membrane barrier. Ultrasound-targeted microbubble destruction (UTMD) is a developed technology that plays important biological roles by the cavitation effect and sonoporation that occurred when ultrasound meets microbubbles, including improving the transfenction efficiency of gene [34, 35], promoting the homing of stem cells [36, 37]. Notably, it has the potential of helping nanoparticles break through the barrier by sonoporation [38]. Nanoparticles and microbubbles were mixed and injected into the body and then followed by ultrasonic irradiation. The microbubbles continuously expanded and contracted and acted on the blood vessel wall or cell membrane, thus increasing the width of endothelial cell gap and the permeability of cell membrane. At the same time, the shock wave and high-speed jet generated instantly when the microbubbles are destroyed, which can also generate large holes in the cell membrane, and help the nanoparticles break through the barrier of entry into the tumor cells [39]. And then the nanoparticles may achieve high concentration of local diffusion and achieve the purpose of targeted therapy in targeted tumor tissue [40].

**Scheme 1:**
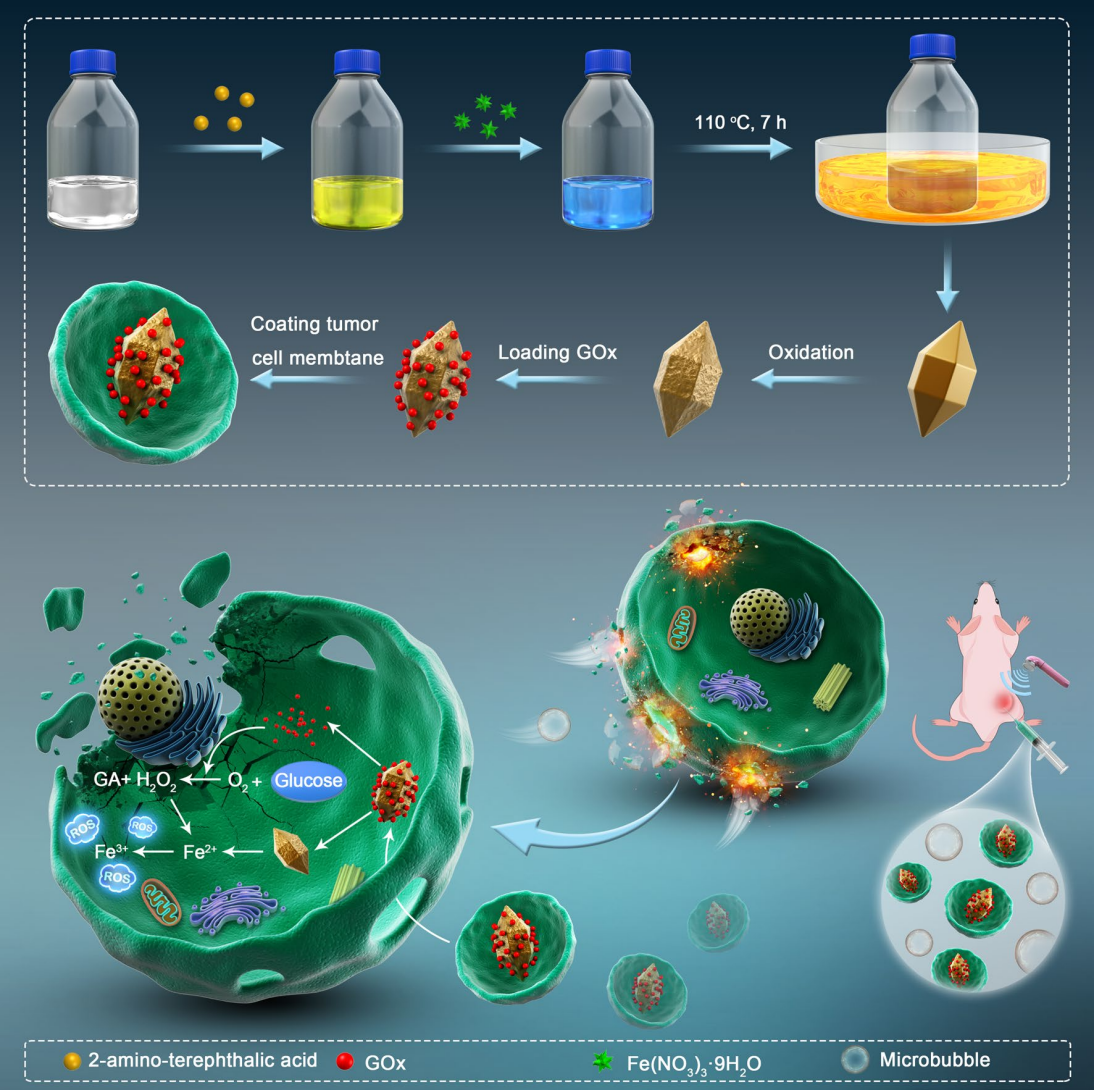
The diagram of FeN200@GOx@M synthesis and anti-tumor principle in vivo

Herein, in this work, a combinational therapeutic approach is presented by using UTMD technology and nanozyme in treating of cancer (Scheme 1). It is expected that such a combination can maximize the catalysis of nanozyme, and effectively realize the anti-tumor effect.

## Results and discussion

**Fig. 1:**
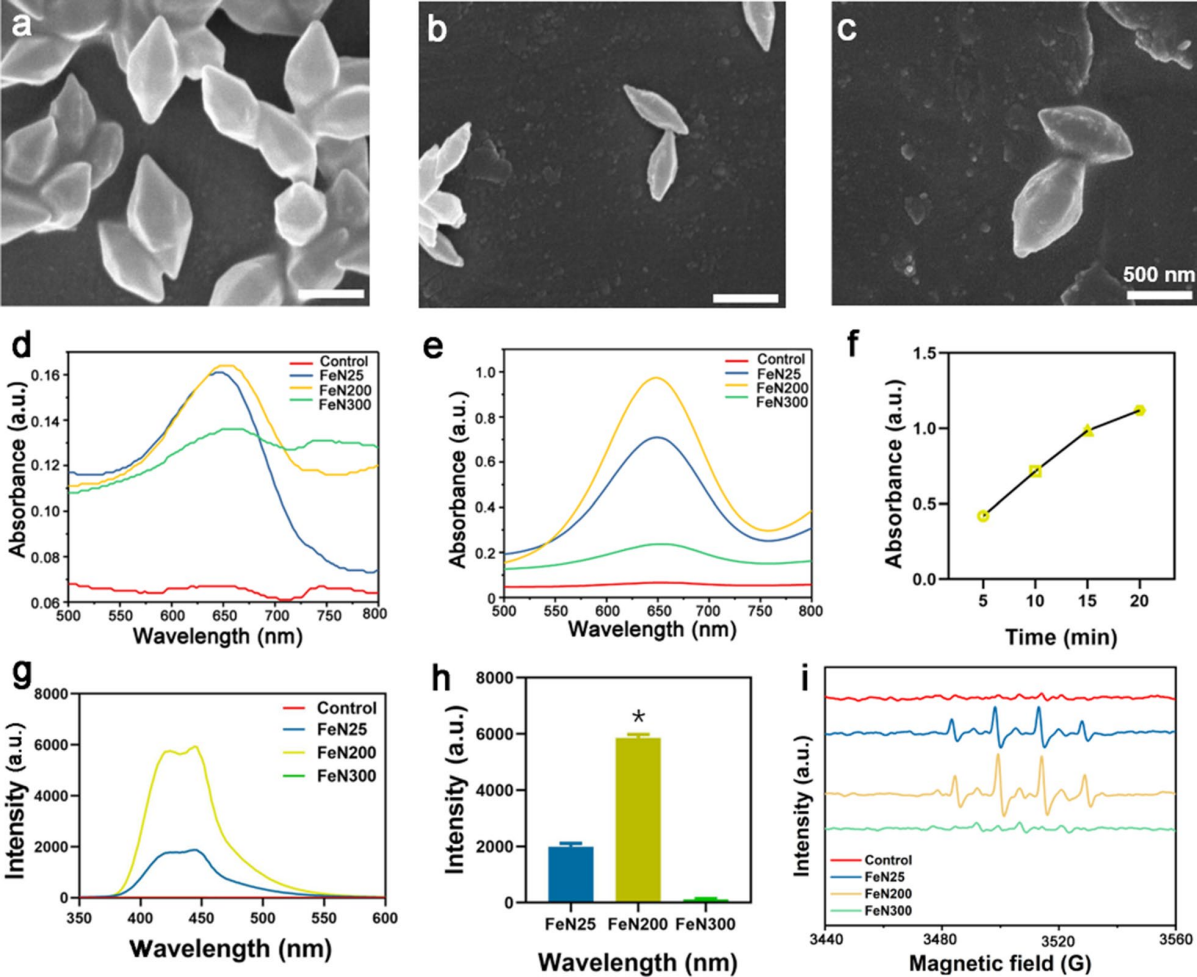
Morphology and catalytic performance of FeNx. SEM images of FeN25 (**a**), FeN200 (**b**), and FeN300 (**c**). **d** OXD activity of three kinds of nanoparticles. **e** POD activity of nanoparticles. **f** POD activity of FeN200 in different time. **g**, **h** TA detection and statistical analysis of nanoparticles. **i** EPR detection of the generation of ·OH in different nanoparticles. The scale bar showed in Figure a-c indicated 500 nm. * indicated P < 0.05

In a typical experiment, spindle Fe-based nanoparticles were first prepared. FeN were modified by oxidation at different temperatures. Since the maximum temperature of oxidation was just 300 °C, the spindle-like morphology of each group of nanoparticles remained stable as observed in scanning electron microscope (SEM) images (Fig. 1a–c). The particle sizes of FeN25, FeN200 and FeN300 were 815.3 ± 12.5 nm, 598.5 ± 14.6 nm and 783.4 ± 20.3 nm, respectively (Additional file 1: Fig. S1), and zeta potentials were − 18.1 ± 1.0, − 17.5 ± 0.8. − 17.7 ± 0.6, respectively (Additional file 1: Fig. S2). Obviously, FeN200 had a relatively smaller size and similar zeta potential when compared with FeN25 and FeN300, which indicated better application prospects. The powder X-ray diffraction (XRD) pattern of FeN (Additional file 1: Fig. S3) revealed that crystals in the obtained products were Fe_3_O_4_. Since Fe_3_O_4_ and Fe_2_O_3_ show very similar XRD patterns, X-ray photoelectron spectroscopy (XPS) for further analysis of Fe chemical status proved that the characteristic peaks of 711.0 and 713.3 eV were corresponding to Fe 2p_1/2_ and Fe 2p_3/2_ (Additional file 1: Fig. S4), supporting that both Fe^2+^(52%) and Fe^3+^(48%) existed in iron oxide and confirming that Fe_3_O_4_ was formed in the nanoparticles.

The catalytic performance of FeN25, FeN200 and FeN300 varied with the oxidation temperature. For the oxidase (OXD) activity, FeN25 and FeN200 oxidized tetramethylbenzidine (TMB) slightly better than FeN300 on the condition of no H_2_O_2_ (Fig. 1d). While for the peroxidase (POD) activity, FeN200 exhibited superior performance with a high absorbance at wavelength of 652 nm. And the absorbance peak increased with the extension of reaction time (Fig. 1e, f). Moreover, terephthalic acid (TA) was used to track the capture of ·OH and generate 2-hydroxy terephthalic acid, along with emitting unique 435 nm fluorescence light (Fig. 1g), and the result showed the intensity of FeN200 group after interaction with TA was significantly higher than FeN25 and FeN300 (Fig. 1h, P < 0.05). To further determine the ability of FeNx nanoparticles to produce ROS, electron paramagnetic resonance (EPR) was adapted to detect the generation of ·OH. Compared with FeN25 and FeN300, FeN200 showed an extremely higher ·OH generation efficiency (Fig. 1i). Combined with the catalytic performance tests of FeNx synthesized under different temperature, FeN200 has the strongest ROS production ability, and has great potential in destroying the intracellular balance of tumor cells and inducing tumor cell apoptosis.

**Fig. 2:**
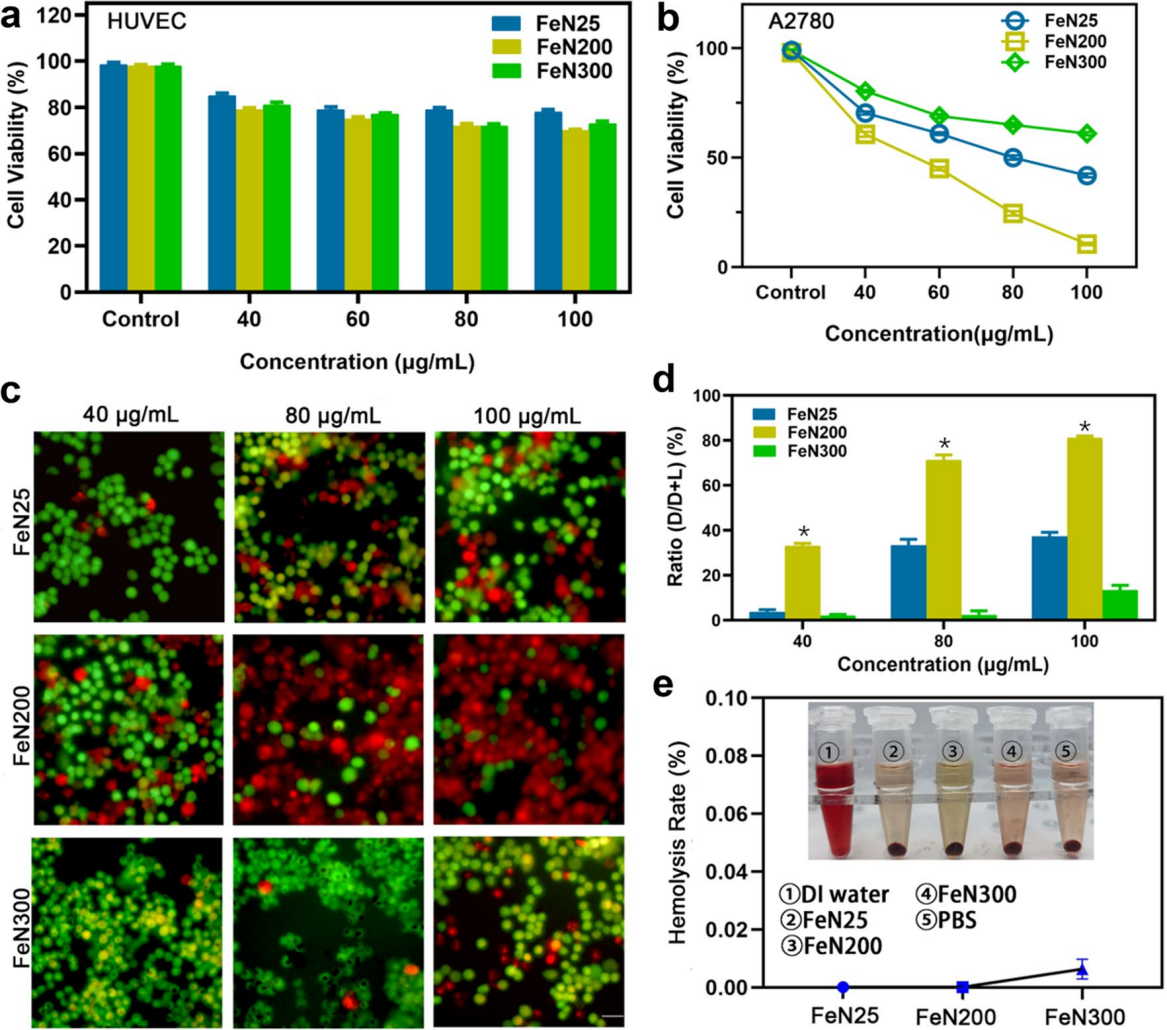
Efficacy and safety evaluation in vitro. **a** Cell viability of HUVEC detected by CCK-8 kit without statistical difference (P > 0.05). **b** Cell viability of A2780 tumor cells detected by CCK-8 kit. **c** Cytotoxicity of nanoparticles in different concentration, the green fluoresced cells indicated the live cells while red fluoresced cells presented the dead cells. **d** Quantitative analysis of fluorescence intensity. D: dead cells; L: live cells. **e** Hemolysis experiment of each nanoparticles. DI water: deionized water. * indicated P < 0.05

To evaluate whether FeNx can be safely and effectively used for cancer therapy in vitro, we carried out a series of experiments to demonstrate it. First, we examined their potential toxicity for biomedical application. Human umbilical vein endothelial cells (HUVECs) and A2780 ovarian tumor cell were cultured respectively, followed by adding different concentration of FeNx. CCK-8 kit results revealed that the cell viability of HUVECs were just slightly decreased as the increase of concentration of FeNx without statistical difference (P > 0.05), and the cell viability could maintained over 70% even at the highest concentration (100 μg/mL) (Fig. 2a). While in the tests on A2780 cells, cell viability decreased obviously with the increase of FeNx concentration. Among the three nanoparticles, FeN200 inhibited the cell viability of A2780 significantly (Fig. 2b). Those results initially demonstrated that the FeNx have potential toxicity for tumor cells instead of healthy cells, and FeN200 had the highest toxicity. The cytotoxicity results could be further visualized by inverted fluorescence microscope imaging of treated cancer cells which were co-stained with FDA working solution (viable cells staining, green fluorescence) and PI (dead cells staining, red fluorescence). A2780 tumor cells treated with FeN200 exhibit a major red fluorescence in contrast to the FeN25 and FeN300 groups in each concentration of nanoparticles (Fig. 2c-d). For nanoparticles that enter the human body or come into direct contact with blood, blood compatibility is a very important safety evaluation index. If endovascular hemolysis occurs, it will cause serious toxicity. Compared with the obvious hemolysis in the deionized water (DI water) as a positive control, no visual hemolysis was found with FeNx, even at a high concentration of 2 mg/mL, showing great comparability with the negative control (phosphate-buffered saline, PBS) (Fig. 2e). These results demonstrated that the FeN200 nanoparticles exhibited excellent cytotoxicity on tumor cells while mild toxicity on HUVECs, and have good biocompatibility and safety for further experiments in vivo.

In this study, FeN200 was selected for further verification based on the catalytic performance and effectiveness of different nanoparticles. Subsequently, GOx was loaded in FeN200 and ovarian cancer cell membranes were finally encapsulated. And finally, FeN200@GOx and FeN200@GOx@M were obtained.

**Fig. 3:**
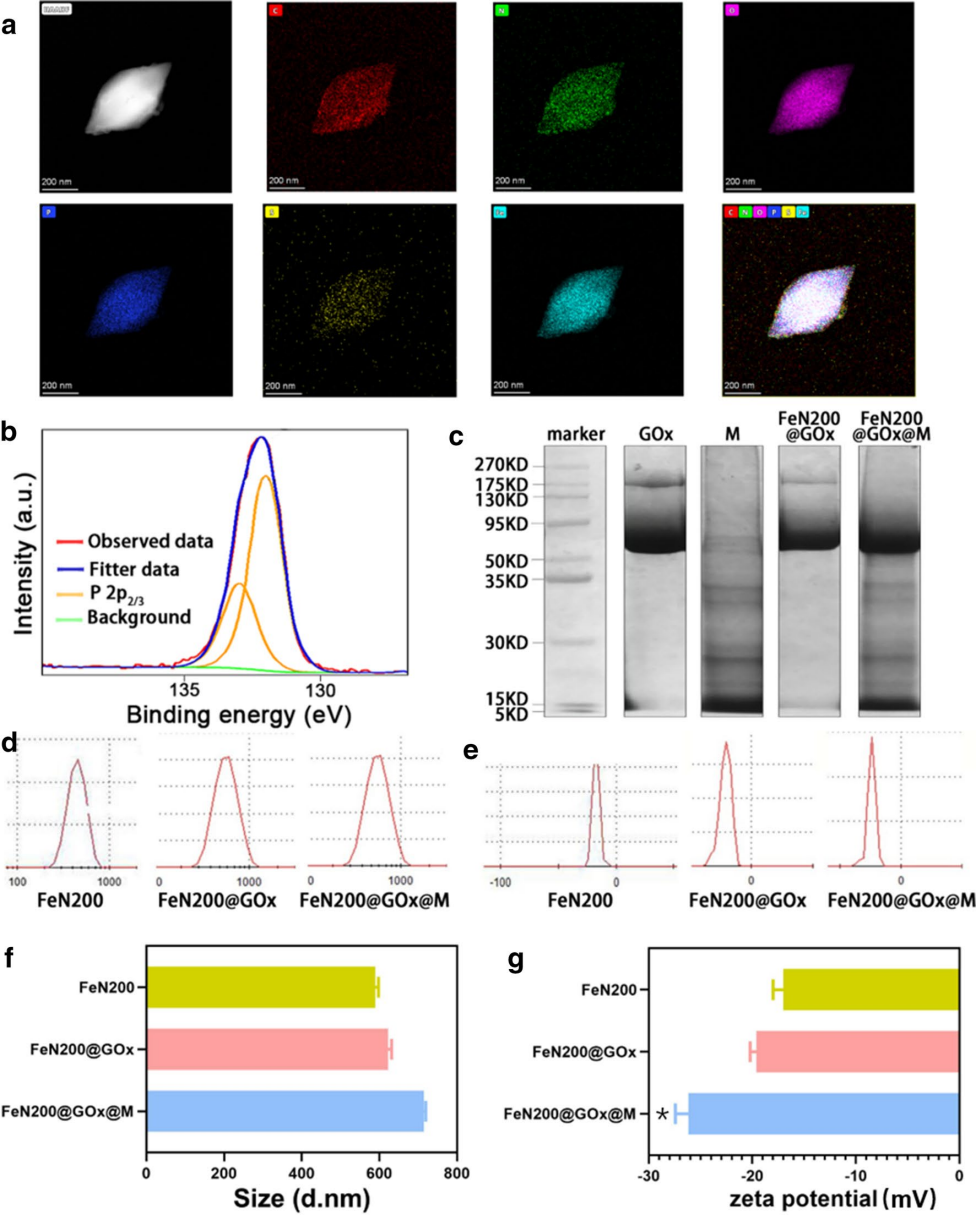
Characteristics of FeN200@GOx@M. **a** The element mapping of FeN200@GOx@M. **b** XPS analysis of phosphorus atoms, indicating the existence of cell membrane. **c** SDS-PAGE imaging revealed the successful loading of GOx and cell membrane on FeN200@GOx@M with corresponding bands. **d**,** f** The average size of different agents. **e**,** g** The average zeta potential of different agents. * indicated P < 0.05

Transmission electron microscopy (TEM) images revealed the high dispersity of FeN200, FeN200@GOx and FeN200@GOx@M and their morphology of bipyramidal hexagonal prism (Additional file 1: Fig. S5). Neither loading GOx nor encapsulating cell membrane had significant effect on the morphology of the nanoparticles. To verify the successful loading of GOx and membrane coating, the following tests were carried out. The element mapping of energy dispersive X-ray spectroscopy (EDS) characterization shown in Fig. 3a and Additional file 1: Fig. S6 demonstrated that elements, including C, N, O, P, S, and Fe, uniformly distributed in FeN200@GOx@M. While XPS spectrums further evidence that the existence of phosphorus atoms (Fig. 3b). What’s more, the results of sodium dodecyl sulfate polyacrylamide gel electrophoresis (SDS-PAGE) indicated that FeN200@GOx had the characteristic band of GOx, and FeN200@GOx@M had the both of the characteristic bands of GOx and tumor cell membrane (Fig. 3c). These results confirmed the successful loading of GOx and membrane coating of FeN200@GOx@M. In addition, the size of FeN200@GOx@M was slightly larger than FeN200 and FeN200@GOx without significant difference (Fig. 3d, f, P > 0.05). All of the three kinds of nanoparticles had good dispersion with dispersibility index (DPI) of 0.127 (FeN200), 0.174 (FeN200@GOx) and 0.270 (FeN200@GOx@M), respectively. And the zeta potential of FeN200@GOx@M was also the highest among the nanoparticles (P < 0.05) (Fig. 3e, g), revealing the satisfying stability of FeN200@GOx@M. The size of the continuous measurements also confirmed the stability of FeN200@GOx@M (Additional file 1: Fig. S7).

**Fig. 4:**
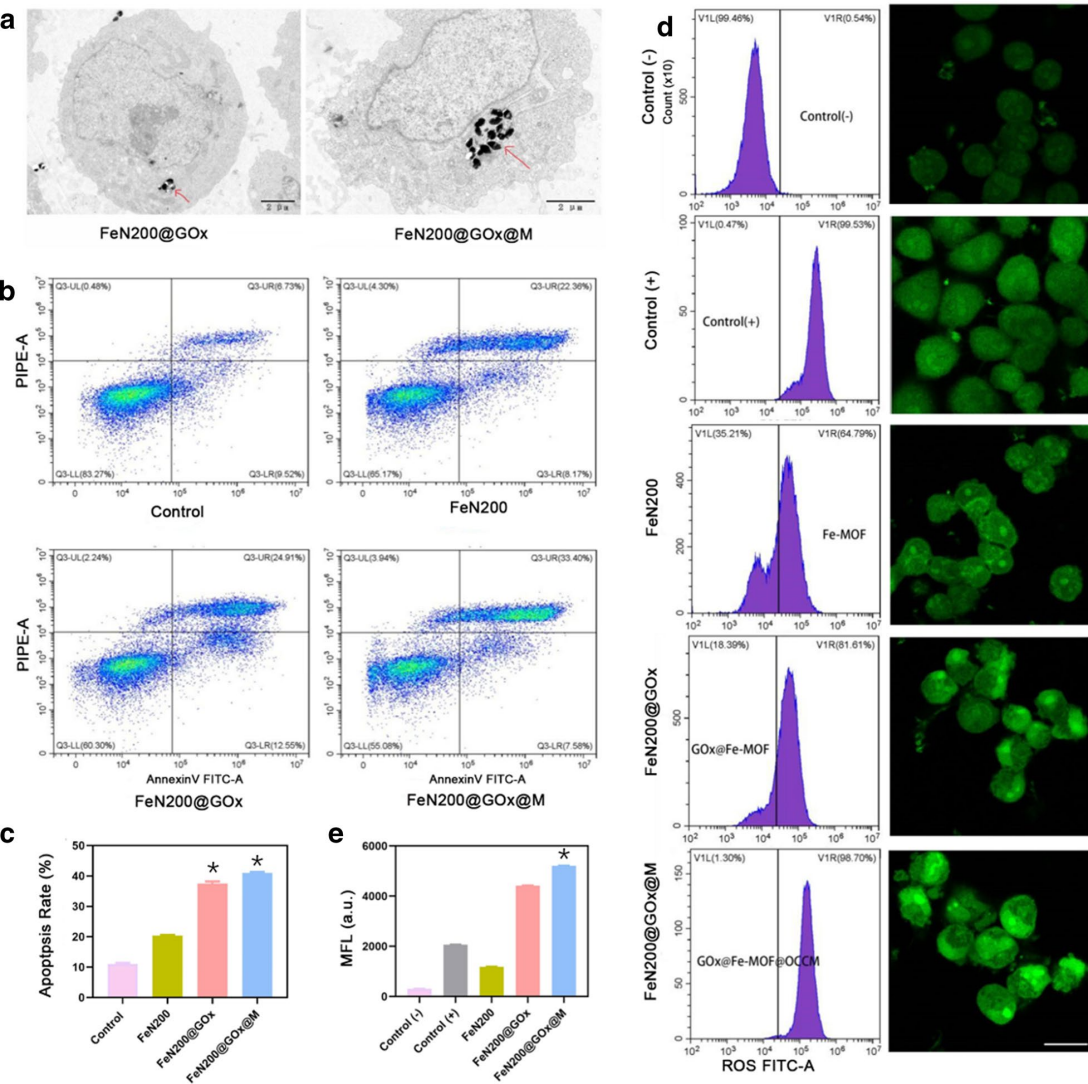
Anti-tumor performance of FeN200@GOx@M in vitro. **a** Bio-TEM showed the intake of nanoparticles in A2780 tumor cells. **b** Flow cytometry indicated the apoptosis of tumor cells when co-incubated with different nanoparticles. **c** The analysis of apoptosis rate in different groups. **d** Flow cytometry and laser confocal microscopy revealed the ROS generation of different nanoparticles. **e)** Quantitative analysis of flow cytometry, MFL: mean fluorescence intensity. * indicated P < 0.05

To further explore the distribution of nanoparticles in A2780 ovarian cancer cells before and after tumor cell membrane coating, bio-TEM was used to assess the intake of nanoparticles. As shown in Fig. 4a, the intracellular FeN200@GOx@M content was significantly higher than that of FeN200@GOx after co-culture of nanoparticles with A2780 tumor cells. The same results were obtained by flow cytometry and its quantitative analysis (Additional file 1: Fig. S8). CLSM exhibited higher fluorescence intensity in A2780 cells than H1299 cells when co-incubation with fluorescence-labeled FeN200@GOx@M (Additional file 1: Figs. S9–S10), indicating that FeN200@GOx@M had certain selectivity of A2780 cells and could be uptake by A2780 cells more easily. The anti-tumor performance of nanoparticles in vitro was evaluated mainly by testing the apoptosis of tumor cells and intracellular ROS production. Annexin V/PI kit was used to assess apoptosis in A2780 tumor cells after co-incubation with nanoparticles by flow cytometry. The results showed that the apoptosis rates of FeN200@GOx@M and FeN200@GOx were higher than the other groups (P < 0.05), and FeN200@GOx@M exhibited the highest apoptosis rate of (41.0 ± 0.3)%, FeN200@GOx, FeN200, and blank control presented an apoptosis rate in descending order (Fig. 4b, c). Meanwhile, DCHF-DA was applied to measure the ROS level in cells, and both of flow cytometry and laser confocal microscopy were used to observe the ROS generation. ROS activates apoptosis signaling pathways such as lysosomes and mitochondria, causing apoptosis of tumor cells [41]. Therefore, the higher the ROS level, the better the antitumor effect. In this study, both of flow cytometry and laser confocal microscopy exhibited that FeN200@GOx@M had the most ROS generation (Fig. 4d). Quantitative analysis by flow cytometry revealed the mean fluorescence intensity (MFL) of FeN200@GOx@M was (5207.5 ± 5.4), which was significantly higher than the other groups (P < 0.05) (Fig. 4e). In addition, cytoskeletal change and intracellular mitochondrial morphology change can also reflect the killing efficiency on tumor cells. Mitochondria are also involved in ROS synthesis, inflammatory response, cell death and other biological processes. They are important suborganelles that regulate cell apoptosis and can be used as important targets to induce apoptosis of tumor cells [42, 43]. Excessive ROS can affect mitochondrial morphology and function [44, 45]. In the control group, the microfilaments were the stress fibers running through the whole length of the cells, while in experimental groups, especially in FeN200@GOx@M group, the cells have lost their microfilaments, which were “ring” shaped and spread across the cell membrane (Additional file 1: Fig. S11). The intracellular mitochondrial morphology was destroyed most obviously in FeN200@GOx@M group among all groups (Additional file 1: Fig. S12). The above results indicated that FeN200@GOx@M could more easily get intake by tumor cells in vitro, and play a better role in ROS generation, apoptosis induction, and finally reach the purpose of anti-tumor compared with other nanoparticles.

Biomimetic nanoparticles camouflaging with natural cell membranes have attracted more and more attention due to their good biocompatibility, good targeting and low immunogenicity [23]. In this study, GOx and Fe-based MOF formed the basis of nanoparticles, thus H_2_O_2_ could be transformed into ·OH with stronger action through cascade catalysis, and tumor cells could be killed by Fenton reaction induced [46].

FeN200@GOx@M exhibited good performance of anti-tumor in vitro, however, the size was not so satisfying due to the obstacles of vascular endothelial cell barrier and membrane barrier. UTMD technology can help the nanoparticles break through the barrier of cell membrane by sonoporation, which means creating large holes in the cell membrane reversibly [39]. And the high concentration of nanoparticles can be achieved by local diffusion and targeted therapy can be realized in target tissue [40]. The combination of UTMD and nanoparticles have the bio-/enzyme-mimics nanoparticles is expected to achieve better anti-tumor effects.

**Fig. 5:**
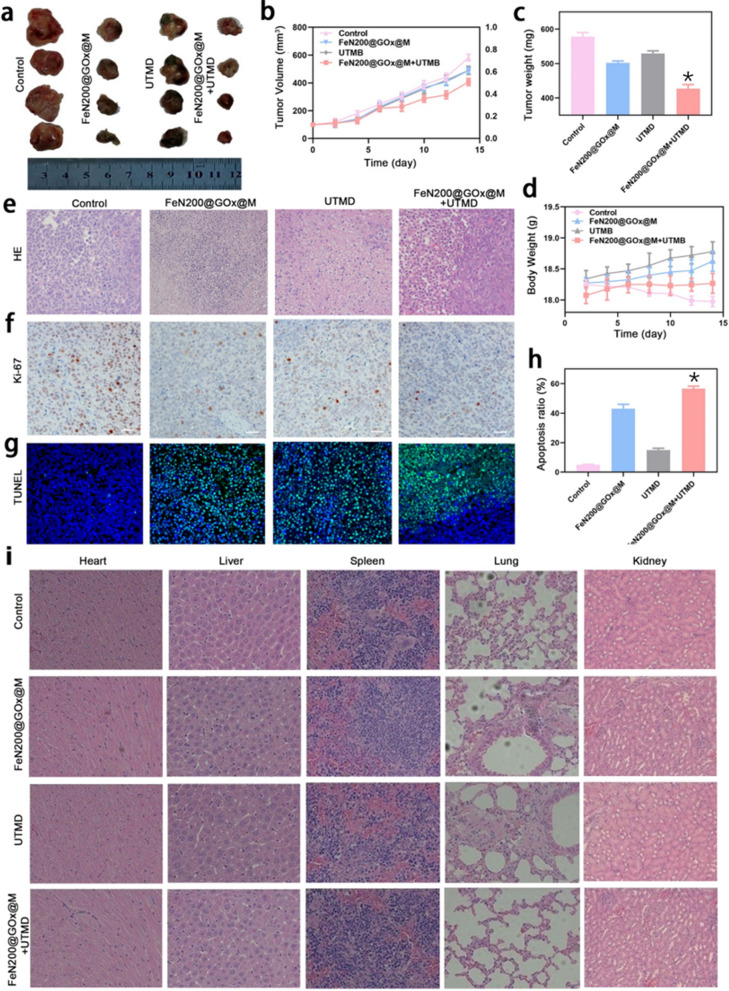
Tumor therapy based on FeN200@GOx@M combined with UTMD in vivo. **a** A general review of the excised tumor in different groups. **b** Tumor volume and **c** tumor weight of the tumors in all groups. **d** Body weight of nude mice in all groups during the treatment. **e** HE staining, **f** Ki-67 immunohistochemical staining, and **g** TUNEL detection of the tumors in all groups. **h** Quantitative analysis of apoptosis ratio. **i** HE staining of vital Organs, including heart, liver, spleen, lung, and kidney in all groups. * indicated P < 0.05

To evaluate the ability of the combination of UTMD and FeN200@GOx@M to destroy tumor cells in vivo, we employed an animal model using BALB/c nude mice bearing A2780 tumor cells. FeN200@GOx@M and microbubble were intratumorally injected (when the tumor size meets the experimental requirements), and the tumor progress was monitored using portable ultrasound (Additional file 1: Fig. S13). Weight changes in each group were also recorded during treatment. After 2 weeks of treatment, tumor tissues of each group were completely exfoliated, tumor volume was measured and tumor weight was weighed. As shown in Fig. 5a, the size of tumors in both of FeN200@GOx@M and UTMD groups shrank to varying degrees visually. Notably, the FeN200@GOx@M + UTMD group had the smallest tumor size among all four groups. Measurements of tumor volume and weight also confirmed that the FeN200@GOx@M + UTMD group had the largest reduction in tumor size (P < 0.05) (Fig. 5b, c). In addition to considering the anti-tumor effect of different interventions, potential advance reactions should also be considered during the treatment process, which can be directly reflected by changes in body weight of nude mice. As exhibited in Fig. 5d, the body weight of the nude mice in control group decreased slightly, while it did not decrease significantly in other groups under the condition of receiving common diet.

In order to further verify the anti-tumor effect from the perspective of pathological changes, HE staining, Ki-67 immunohistochemical staining and TUNEL detection were performed on the removed tumor tissues. As shown in Fig. 5e, the tumor cells were distributed diffusely and patchy, and the nuclei were intact in control group. While the number of tumor cells decreased to some extent when receiving different treatment, and FeN200@GOx@M + UTMD group had the most severe tissue damage, indicating that the growth of tumor cells was obviously inhibited. Ki-67 antigen, a nuclear antigen associated with proliferating cells, is associated with cell mitosis and is a reliable marker of tumor cell proliferation. The positive number of cells (claybank granules) in FeN200@GOx@M + UTMD group was significantly less than the other groups (Fig. 5f). TUNEL staining, mediated by terminal deoxynucleotide transferase, can directly label apoptotic cells in tumor tissues after treatment. The bright green granules presented in Fig. 5g indicated the apoptotic tumor cells. Quantitative analysis of tumor cell apoptosis showed that the apoptosis rate was the highest in FeN200@GOx@M + UTMD group (Fig. 5h, P < 0.05). In addition, HE staining was performed on the vital organs of each group, including heart, live, spleen, lung, and kidney, respectively, to determine whether the treatment strategy had adverse influence on the body. The results revealed that there were no significant abnormalities in the vital organs in each group, suggesting that the treatment involved in this study only had a therapeutic effect on the local tumor (Fig. 5i).

Together, these animal experiments demonstrated that with the sonoporation of UTMD, FeN200@GOx@M successfully acted on tumor cells in vivo, then uniformly disperse in the tumor regions and shrink the tumor tissues, thus effectively treating the tumors and have potential for improving the survival rate of tumor bearing mice.

## Conclusion

In summary, we designed a Fe-MOF based bio-/enzyme-mimics nanoparticle for the treatment of cancer. FeN200@GOx@M has excellent ROS generation performance under physiological conditions and shows satisfying anti-tumor properties in vitro. The combination of FeN200@GOx@M and UTMD can further effectively increase the inhibition of tumor growth in vivo. Taken together, we believe the bio-/enzyme- mimics nanoparticle generating ROS will have great potential in more diseases, and UTMD adds to its therapeutic efficiency notably.

## Materials and methods

### Fe-MOF based nanoparticles fabrication

79 g 2-amino-terephthalic acid was dissolved in 100 mL *N*,*N*-Dimethylformamide (DMF), followed by adding 3.99 g Fe(NO_3_)_3_·9H_2_O and stirring until dissolved. The reactants were heated in 110 °C oil bath for 7 h. The precipitate obtained by centrifugation n (5000 rpm, 5 min) was washed alternately with DMF and ethanol for three times and then dried to obtain the Fe-MOF based nanoparticles (FeN). Then the FeN were placed in a tubular furnace and oxidized for 2 h at different temperatures of 25 °C, 200 °C and 300 °C, with the temperature rising by 5 °C per minute. The corresponding products were called FeN25, FeN200 and FeN300.

### Characterization of FeN

The morphology of FeN was observed by SEM and the crystal morphology was checked by XRD. In order to determine the valence state of Fe, XPS was also performed.

### Catalytic activity measurements of FeN

TMB was employed to measure OXD and POD activities of FeN. Briefly for OXD activity, 24 μL TMB (10 mg/mL, DMF) and 10 μL FeN solution (4 mg/mL) were added into 2 mL PBS. For POD activity, 24 μL TMB (10 mg/mL, DMF), 20 μL H_2_O_2_ and 10 μL FeN solution (4 mg/mL) were added into 2 mL PBS. After 10 min, the reactants were centrifuged instantaneously and the absorption spectra of the supernatant were continuously scanned by a microplate reader.

### Hydroxyl radical evaluation

TA can produce detectable fluorescence when oxidized by ·OH, so it is often used to evaluate the production of ·OH. Typically, 300 μL FeN (100 μg/mL), 300 μL TA (5 mM), 300 μL H_2_O_2_ (1 mM) and 300 μL PBS (0.01 M, pH = 7) were mixed and diluted to a total volume of 3 mL by DI water. After darkly bathed in 30 °C water for 12 h, the reactants were centrifuged instantaneously and the absorption spectra of the supernatant were continuously scanned by a fluorometer (excitation wavelength 315 nm, emission wavelength 340–625 nm, peak wavelength 435 nm). DMPO was employed as the nitrogen trap for ·OH to form DMPO-OH adducts for detection in EPR experiment. Briefly, DMPO (50 mM) was added into the buffer system containing H_2_O_2_ (50 μM) and FeN 2 mg/mL, 50 μL). Then an aliquot of the solution was transferred to a quartz tube for EPR essay after violent shaking for several seconds.

### In vitro* cell-viability assays and staining*

5 × 10^3^ umbilical vein endothelial cells (HUVECs) and A2780 tumor cells suspended in 100 μL 1640 medium per well were inoculated into the 96-well plates respectively. Cells were incubated overnight for adhering. Then discard the previous medium and wash the cells with PBS twice. The FeN (40, 60, 80, 100 μg/mL) and H_2_O_2_ (100 μM) suspended in 100 μL fresh 1640 medium were supplemented. After 20–30 min co-incubation with cells, a typical colorimetric cell-counting kit-8 (CCK-8) assay was added to assess to cell viabilities according to the protocol. The absorbance was read on a microplate reader with a filter of 450 nm.

For cell staining, A2780 tumor cells were incubated and adhered in 96-well plate based on the above method. After rinsed with PBS, 100 μL fresh 1640 medium containing with FeN (40, 80, 100 μg/mL) were added and then co-incubated for 12 h. The previous medium was discarded and rinsed with PBS again. Then 100 μL FDA working solution (10 μg/mL) was employed to stain under dark for 5 min, followed by adding 100 μL PI working solution (20 μg/mL) for another 5 min in dark. Finally, the staining solution were removed and rinsed with PBS twice gently afterward prior to the inverted fluorescence microscope observations.

### Hemolysis test

500 μL 2% sheep red blood cells (SRBC) and 500 μL FeN solution (2 mg/mL) were mixed and heat preservation in 37 oC water bath for 3 h. DI water was used for the positive control and PBS for the negative control. After centrifugation at 1000 g for 10 min, the supernatant was taken to test the absorbance at 545 nm. The hemolysis rate was calculated by the following equation.

Hemolysis rate = (OD_FeN_− OD_negative control_)/( OD_positive control_ − OD_negative control_) × 100%

### Fe-MOF based bio-enzyme-mimics nanoparticles fabrication

The optimal FeNx was selected by the above tests. 25 mg FeNx, 25 mg GOx and 25 mL PBS were mixed, and ultrasonic dispersion was performed for 15 min under ice bath. Then the solution was dried by vacuum freeze evaporation at − 80 °C. After complete drying, 25 mL DI water was added for resuspended suspension and centrifuged (5000 rpm, 5 min) to remove the free GOx and collect the supernatant and precipitation respectively. Then the precipitation was freeze-dried at -80 °C again, and FeNx@GOx was obtained. BCA protein concentration determination kit was used to evaluate the content of GOx in supernate.

Enzyme lysis and ultrasonic crushing were used for the successful extraction of tumor cell membrane according to Han’s research [47]. FeNx@GOx and cell membrane with mass ratio of 1:1 was added in an EP tube for 30-min ultrasonic crushing on ice [46]. Blow the solution gently every 5 min for homogeneous dispersion. Finally, excess cell membrane fragments were removed by centrifugation (10,000 rpm, 10 min), and FeNx@GOx@M nanoparticles were obtained.

### Characterization of FeNx@GOx@M

TEM was employed for the observation of morphology of nanoparticles. The EDS and XPS were used to identify the successful encapsulation of cell membrane. To further verify the loading of GOx and cell membrane, SDS-PAGE was used. Briefly, after the electrophoresis of different proteins in the prefabricated gel, Coomace bright blue solution was used to dye the gel and then discolored. Finally, the protein gel was photographed for analysis.

The size and zeta potential of FeN200, FeN200@GOx and FeN200@GOx@M were assessed by Nano-ZS90 Laser nanometer. To evaluate the stability of FeN200@GOx@M, the particle size was continuously observed on day 0, 1, 2, 3, 4, 5, 6, 7, respectively.

### Intake of particles in A2780 tumor cell

1 × 10^6^ A2780 tumor cells suspended in 1 mL 1640 medium were inoculated into a 3 cm culture dish for 24 h incubation. Then replace the previous medium of fresh medium containing different particles (20 μg/mL). After 4 h co-incubation, the cells were rinsed with PBS, then digested with trypsin, centrifuged and washed with PBS for 3 time, and then collected. Finally, the 2.5% glutaraldehyde fixative was added gently and observed the cells with TEM.

Flow cytometry was used to quantify the uptake behavior of cells. FeN was labeled with fluorescence by adsorbing DiI fluorescent dye before the experiment. Briefly, the cells were treated the same way as before. After digestion with trypsin and collection, the cells were resuspended with 150 μL PBS and detected by flow cytometry. Cytexpert software was used to analyze data.

To verify the possible selectivity of nanoparticles to different tumor cells, Dil-labelled FeN200@GOx@M was co-incubated with A2780 cells and H1299 cells respectively. Finally, the cells were observed under CLSM, and corresponding fluorescence intensity was analyzed.

### *Apoptosis of A2780 tumor cell *in vitro

1 × 10^6^ A2780 tumor cells were inoculated into a 6-well plate for 24 h incubation. Then replace the previous medium of fresh medium containing FeN200, FeN200@GOx and FeN200@GOx@M (20 μg/mL), respectively. After 12 h co-incubation, carefully collected the culture medium into a 5 mL centrifuge tube. Cells were digested with trypsin without EDTA and collected again into the centrifuge tube. Then centrifuged the solution to precipitate (1000 rpm, 5 min). The obtained cells were resuspended with a Binding buffer to adjust the concentration to 5 × 10^6^ cell/mL. 100 μL cell suspension was placed in a 1.5 mL EP tube, then 5 μL Annexin-V-FITC was added in dark and 10 μL PI solution was added 5 min later. Finally, flow cytometry was performed within 10 min after adding 400 μL Binding buffer.

### *ROS generation of nanoparticles *in vitro

The cells were cultured in the same way as 2.10 except for that cells were incubated in 3 cm confocal culture dish instead of 6-well plate. Besides the FeN200, FeN200@GOx and FeN200@GOx@M groups, negative control (medium) and positive control (stimulant in ROS kits) were also set. After 12 h co-incubation, 500 μL DCFH-DA solution (10 μM) were added for further 30 min incubation. Then discard the previous medium and add 1 mL fixation solution for 24 h. Finally, the cells were sealed and observed by laser confocal microscopy.

Flow cytometry can evaluate the generation of ROS quantitatively. After co-incubation with DCFH-DA solution, trypsin was used to digest and collect the cells. Finally, 150 μL PBS was employed to resuspend cells for flow cytometry test.

### *Mitochondrial morphology and cytoskeleton detection *in vitro

Mito-tracker Green fluorescent probe (working solution concentration 100 nM) was used to reveal mitochondrial morphological changes in A2780 tumor cells after co-incubated with FeN200, FeN200@GOx and FeN200@GOx@M nanoparticles. 0.5% solution of Triton X-100, rhodamine-labeled phalloidin (TRITC phalloidin) working solution (100 nM) and DAPI were employed for the detection of cytoskeleton. The cells were observed under confocal microscope.

### Subcutaneous transplantation cancer model in BALB/c nude mice and experiment procedures

A total of 16 BALB/c nude mice (female, weighed 13–16 g) were used in the experiment, and were separated into four groups randomly. 1 × 10^7^ A2780 tumor cells suspended in PBS (100 μL)were subcutaneously inoculated into the right flank of every nude mouse. When the tumor volume was about 100 mm^3^ (about 2 weeks), grouping treatments was performed as follows: (1) Control; (2) FeNx@GOx@M; (3) UTMD; (4) FeNx@GOx@M + UTMD. 100 μL nanoparticles (1 mg/mL) or SonoVue solution were injected intratumorally according to the grouping. For the control group, 0.9% saline was injected intratumorally instead. The detailed procedures of different groups were shown in Table 1. According to the previous study [48], the in vivo experimental ultrasonic irradiation conditions were set as: frequency 1 MHz, sound intensity 1 W/cm^2^, duty cycle 20%, irradiation time 3 min. The day of the first treatment was recorded as day 0, and the treatment was repeated every other day for 7 consecutive times. The mice were housed in SPF-level animal laboratories. All of the animal procedures were performed in compliance with the guidelines of the Animal Research Committee of Sichuan University.

**Table 1:** Experimental procedures in vivo

Groups	Experimental procedures
(1) Control	100 μL 0.9% saline injected intratumorally
(2) FeN200@GOx@M	100 μL FeN200@GOx@M (1 mg/mL) injected intratumorally
(3) UTMD	100 μL SonoVue solution injected intratumorally, followed by ultrasonic irradiation
(4) FeN200@GOx@M + UTMD	100 μL FeN200@GOx@M (1 mg/mL) and 100 μL SonoVue solution injected intratumorally, followed by ultrasonic irradiation

Weight of mice was recorded and tumor size was measured by portable ultrasound before each treatment. After finishing the last treatment, the mice were sacrificed, the tumors were removed and then tumors’ weight were measured. HE staining, Ki-67 immunohistochemical (IHC) staining and apoptosis testing were performed on the tumor tissues. Meanwhile, the main organs of mice, including heart, liver, spleen, lung and kidney, were taken out for HE staining.

### Statistical analysis

Statistical analysis of all data (mean ± S.D) were performed using SPSS 22.0 (IBM Corp.). One-way analysis of variance and Chi-square test were used for the statistical evaluation. P value < 0.05 was set as the statistical significance level.

## Supplementary Information

**Additional file 1.** Additional Figures S1–S13.

## Data Availability

All data used to generate these results is available within the paper and the Supporting Information.
