# Supplementary material for: Ultrasound targeted microbubble destruction combined with Fe-MOF based bio-/enzyme-mimics nanoparticles for treating of cancer
Source: J Nanobiotechnology. 2021 Mar 31;19:92. doi: 10.1186/s12951-021-00835-2 (PMC8011114; doi:10.1186/s12951-021-00835-2)
Supplement: Supplementary file 1 — Additional file 1. Additional Figures S1–S13. [file 12951_2021_835_MOESM1_ESM.docx]

**Supplementary Information**

**Ultrasound Targeted Microbubble Destruction combined with Fe-MOF based Bio-/Enzyme-Mimics Nanoparticles for treating of Cancer**

Xi Xiang^1#^, Houqing Pang^2#^, Tian Ma^1^, Fangxue Du^1^, Ling Li^1^, Jianbo Huang^1^, Lang Ma^1^, Li Qiu^1^*

^1^ Department of Medical Ultrasound, Laboratory of Ultrasound Imaging Drug, West China Hospital of Sichuan University, Chengdu, 610041, China

^2^ Department of Ultrasound, West China Second University Hospital, Sichuan University/ West China women’s and children’s Hospital, Chengdu, 610041, China

*Corresponding author. E-mail address: qiulihx@scu.edu.cn

^#^These authors contributed equally to this work.


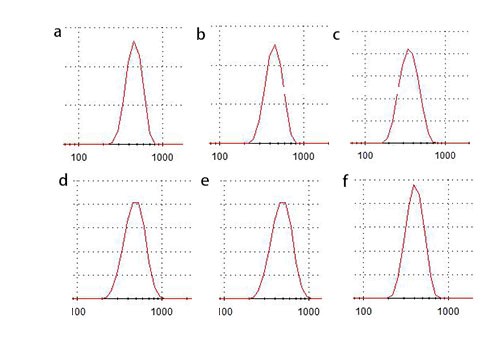


**Figure S1**: The particle sizes of **a** FeN25, **b** FeN200, and **c** FeN300.


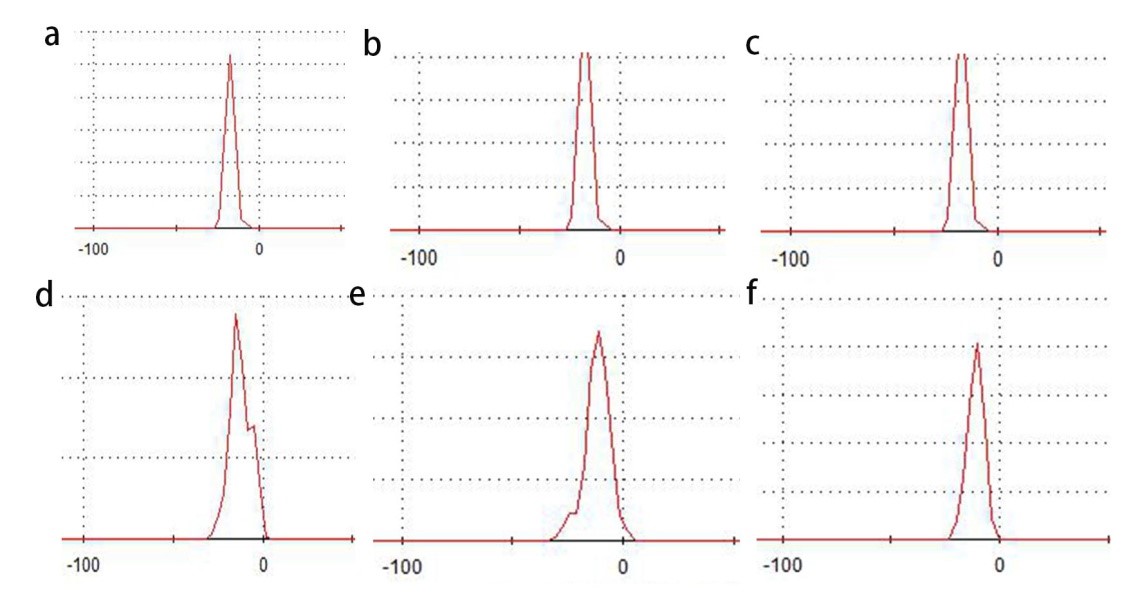


**Figure S2**: The zeta potential of **a** FeN25, **b** FeN200, and **c** FeN300.


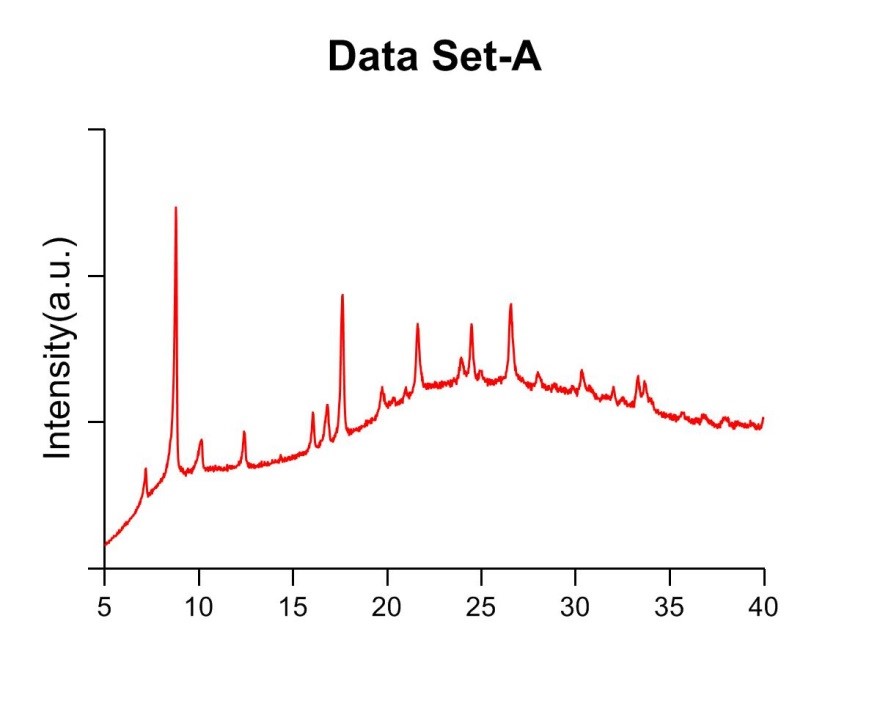


**Figure S3**: XRD pattern of FeN25.


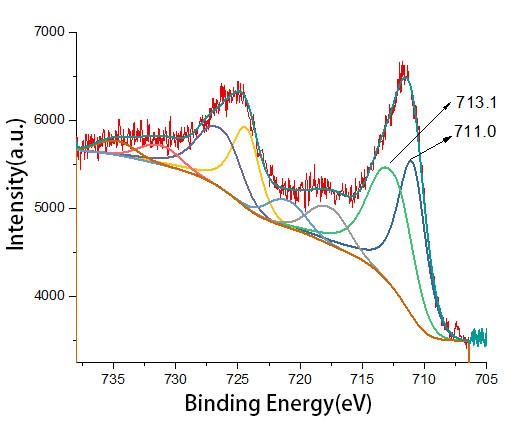


**Figure S4**: XPS analysis of FeN25, supporting that both Fe^2+^(52%) and Fe^3+^(48%) existed in iron oxide and confirming that Fe_3_O_4_ was formed in the nanoparticles.


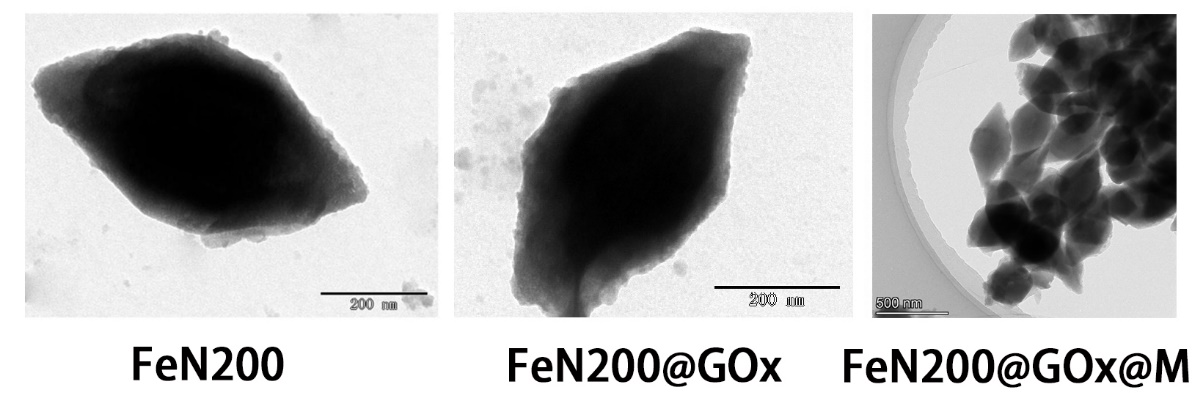


**Figure S5**: TEM images of FeN200, FeN200@GOx, and FeN200@GOx@M.


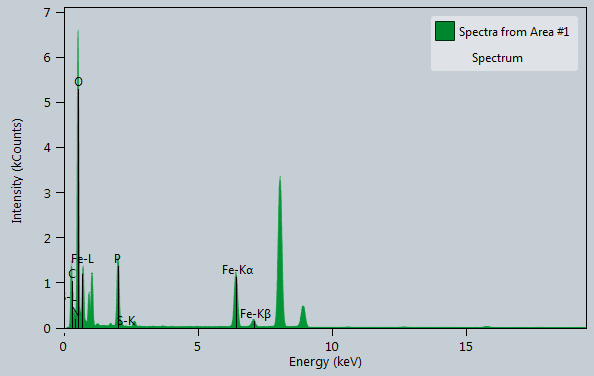


**Figure S6**: EDS analysis of FeN200@GOx@M. P atoms indicated the presence of cell membrane.


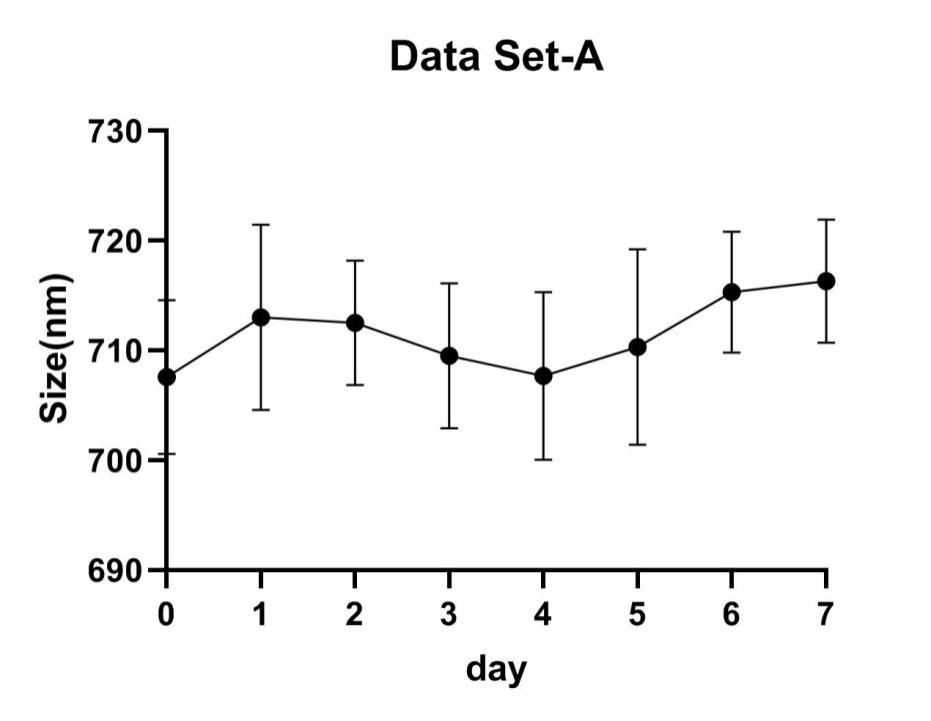


**Figure S7**: The size of FeN200@GOx@M in different time after synthesis, indicating the stability of nanoparticles.


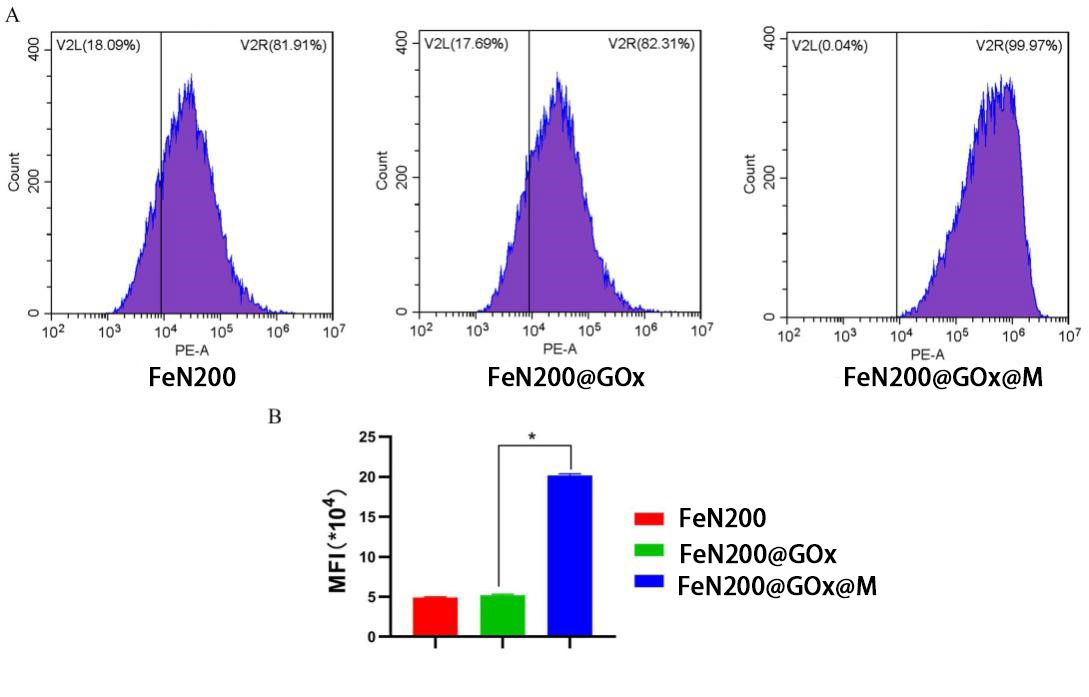


**Figure S8**: Flow cytometry and quantitative analysis of intake of nanoparticles in A2780 tumor cells.


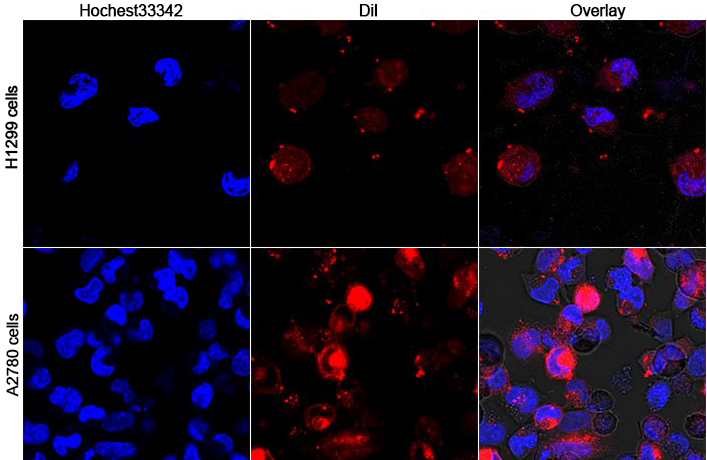


**Figure S9**: The selectivity of FeN200@GOx@M in different cancer cells.


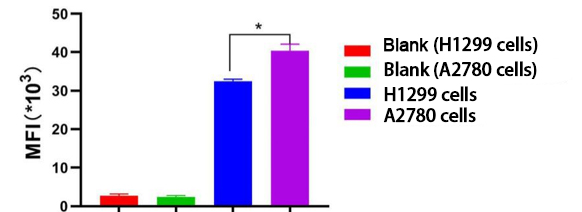


**Figure S10**: The analysis of fluorescence intensity in different cancer cells.


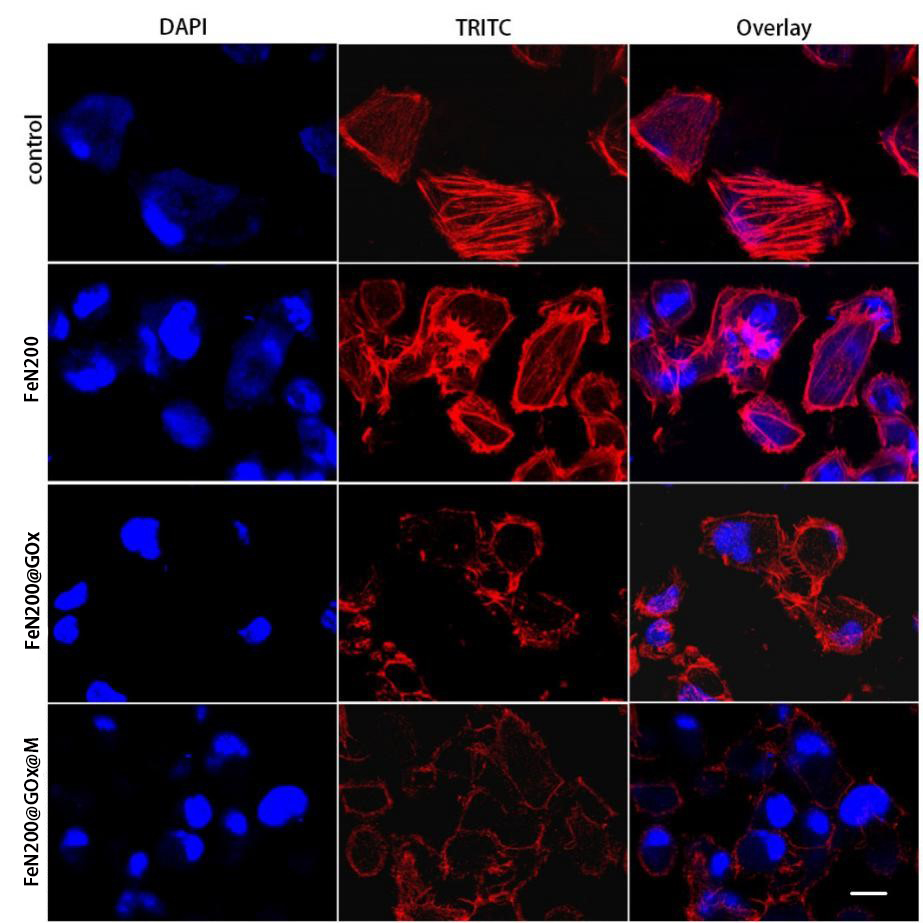


**Figure S11**: Cytoskeletal staining in different groups.


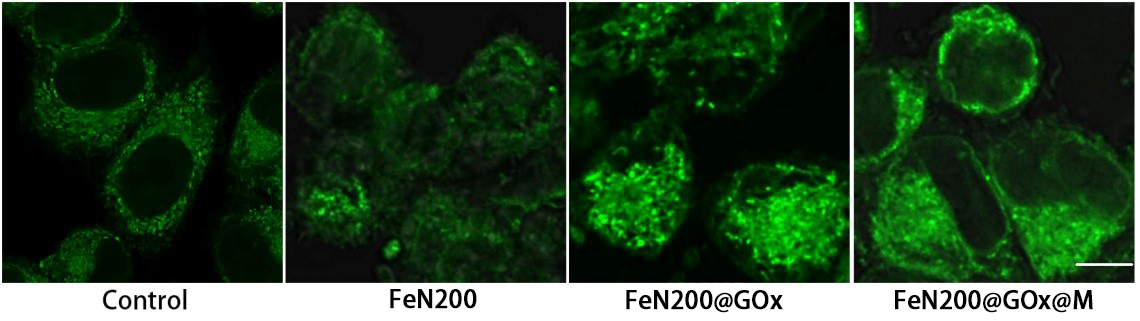


**Figure S12**: Intracellular mitochondrial morphology changes in different groups.


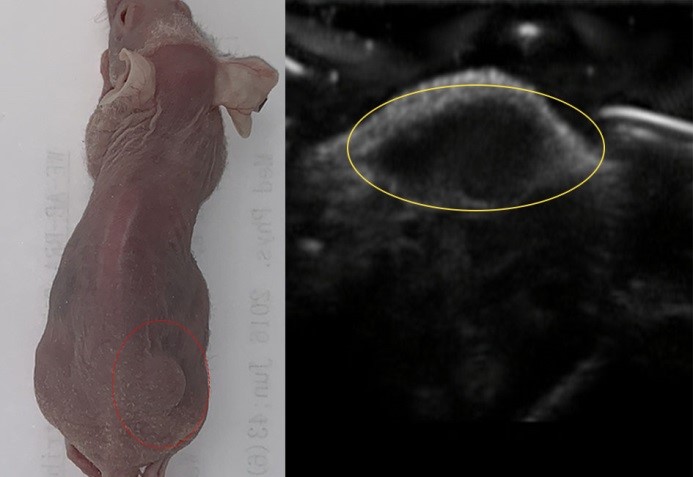


**Figure S13**: A general view of the mouse model and a portable sonogram of the tumor (the yellow circle).
